# Chitosan-Based Cast Films of Different Molecular Weights for Sustained Activity of *Bacillus subtilis*

**DOI:** 10.3390/polym18070784

**Published:** 2026-03-24

**Authors:** Vladimir Krastev, Nikoleta Stoyanova, Iliyana Valcheva, Donka Draganova, Mariya Spasova, Olya Stoilova

**Affiliations:** 1 Laboratory of Bioactive Polymers, Institute of Polymers, Bulgarian Academy of Sciences, 1113 Sofia, Bulgaria; v_krastev@polymer.bas.bg (V.K.); nstoyanova@polymer.bas.bg (N.S.); mspasova@polymer.bas.bg (M.S.); 2 Centre of Competence “Sustainable Utilization of Bio-Resources and Waste of Medicinal and Aromatic Plants for Innovative Bioactive Products” (CoC BioResources), 1000 Sofia, Bulgaria; 3 Biodinamika Ltd., 4000 Plovdiv, Bulgaria; valchevailiana1@gmail.com (I.V.); donkadraganova@gmail.com (D.D.)

**Keywords:** chitosan-based films, *Bacillus subtilis* encapsulation, biocontrol agent, antifungal activity, sustainable plant protection

## Abstract

The development of sustainable plant protection strategies requires stable and environmentally compatible delivery systems for beneficial microorganisms. In this study, *Bacillus subtilis* was encapsulated within chitosan-based cast films to evaluate bacterial viability, sustained biological activity, and antifungal efficacy. Films prepared from chitooligosaccharide (COS) and chitosans of low, medium, and high molecular weight (CS-LMW, CS-MMW, CS-HMW) were characterized in terms of morphology, mechanical performance, and pH-dependent swelling behavior. The viscosity of the chitosan solutions increased markedly with molecular weight from 73 cP (COS) to 614 cP (CS-HMW), while film thickness ranged from 34 ± 1.5 to 57 ± 2.3 µm. Mechanical performance improved significantly with increasing molecular weight, with maximum tensile stress exceeding 200 MPa for CS-HMW films, while swelling studies confirmed pronounced pH-dependent behavior consistent with the polyelectrolyte nature of chitosan. Encapsulation effectively preserved bacterial viability and metabolic activity over time. The intrinsic antifungal activity of chitosan synergized with the biocontrol activity of *B. subtilis* against *Fusarium avenaceum* and *Alternaria solani*. The highest antifungal performance was observed for CS-HMW films, which produced inhibition zones up to 84.6 ± 5.0 mm against *A. solani*. These findings demonstrate that chitosan-based cast films serve as effective carriers for beneficial microorganisms, providing environmental protection and regulated biological activity. The combination of a bioactive polymer matrix with a potent biocontrol agent represents a promising eco-friendly approach to sustainable plant protection.

## 1. Introduction

Plant diseases caused by fungal pathogens represent a major threat to global agricultural productivity and food security. It is estimated that plant pathogens are responsible for approximately 10–16% of global crop losses annually, with fungi accounting for the largest proportion of these losses despite the widespread use of chemical fungicides. In addition, fungal contamination and mycotoxin production affect nearly 25% of the world’s agricultural commodities, leading to substantial economic losses and serious food safety concerns [1,2]. Consequently, the global transition toward sustainable agriculture has intensified efforts to develop environmentally friendly alternatives to synthetic agrochemicals. Biological control agents (BCAs), composed of beneficial microorganisms, offer effective disease management while enhancing plant growth and soil health without causing ecological harm. Among these, plant growth-promoting rhizobacteria (PGPR) are particularly valuable due to their ability to enhance nutrient availability and stimulate plant defenses [3]. *Bacillus subtilis*, a Gram-positive bacterium common in soil and the rhizosphere, is among the most extensively studied PGPR species. Its biocontrol efficacy derives from the production of bioactive secondary metabolites, including cyclic lipopeptides, polyketides, and antimicrobial peptides [4,5,6,7,8]. Beyond direct antagonism, *B. subtilis* induces systemic resistance in plants and outcompetes pathogens for root colonization [9,10]. A key physiological advantage of *B. subtilis* is its ability to form endospores—metabolically dormant, resilient structures that survive heat, desiccation, and UV exposure [11]. Despite this, field application of microbial inoculants remains challenging, as vegetative cells are sensitive to abiotic stress, reducing overall efficacy [12,13].

Encapsulation in biodegradable polymeric matrices protects beneficial microorganisms from environmental stresses, stabilizing their hydration and extending viability [14]. Among naturally derived polymers, chitosan is an ideal carrier due to its biodegradability, cationic nature, intrinsic antimicrobial activity, and film-forming ability [15,16,17,18,19,20,21]. With a pKa ~6.5 [22], chitosan is protonated (and highly soluble) under acidic conditions, whereas deprotonation at neutral pH promotes hydrogen bonding and network consolidation. As a result, molecular weight and chain entanglement density strongly influence mechanical strength, swelling and diffusion properties. Chitosan additionally exhibits intrinsic antimicrobial activity and can elicit plant defense responses, which may synergize with the activity of encapsulated microbes [23,24,25]. Importantly, chitosan cast films form flexible, semi-permeable membranes with good mechanical resistance. These films regulate the diffusion of water, gases, nutrients and metabolites, thereby shaping microbial survival within the matrix [26]. Film casting is a simple, cost-effective, and scalable way to produce such coatings. Film performance—tensile strength, swelling and release behavior, depends strongly on the chitosan molecular weight and degree of deacetylation, which set the network density and transport properties [15,27,28]. Despite these advantages, studies focusing specifically on the encapsulation of beneficial bacteria such as *B. subtilis* into cast chitosan films remain limited.

In previous work, we developed chitosan-coated electrospun poly(3-hydroxybutyrate) (PHB) biohybrid materials as carriers for *B. subtilis*, demonstrating enhanced growth support, improved storage stability, and synergistic antimicrobial effects [29]. Additionally, chitosan gel bead formulations have been shown to promote *B. subtilis* viability and antifungal activity against phytopathogens, highlighting the dual role of chitosan as both a protective carrier and a bioactive enhancer [30]. Based on this prior work, the present study develops and characterizes chitosan-based cast films for the encapsulation of *B. subtilis*. Film mechanical properties and swelling behavior across different pH values are evaluated, with particular emphasis on bacterial viability and sustained activity. The potential synergistic interaction between the intrinsic antifungal properties of chitosan and the biocontrol activity of released *B. subtilis* is assessed against the phytopathogenic fungi *Fusarium avenaceum* and *Alternaria solani*. This study provides insights for the development of robust, environmentally friendly plant protection systems that integrate a bioactive polysaccharide carrier with an effective biological control agent.

## 2. Materials and Methods

### 2.1. Materials

In this study, a chitooligosaccharide (COS) with a viscosity of 50 cPs and a degree of deacetylation of 92.1% was used. Chitosans of varying molecular weights, all with a degree of deacetylation of 91%, were also employed: low-molecular-weight chitosan (CS-LMW, max. 200 cPs), medium-molecular-weight chitosan (CS-MMW, 200–400 cPs), and high-molecular-weight chitosan (CS-HMW, min. 400 cPs). All polysaccharides were obtained from Yantai Shang Tai Trading Co., Ltd. (Beijing, China). Glacial acetic acid and buffer salts of analytical grade were purchased from Merck (Darmstadt, Germany). Buffer solutions at pH 4.0 (CH_3_COOH/NaOH), pH 7.0 (KH_2_PO_4_/Na_2_HPO_4_), and pH 9.0 (NaHCO_3_/Na_2_CO_3_) were freshly prepared and used in the experiments.

*Bacillus subtilis* was obtained from the collection of Biodinamika Ltd. (Plovdiv, Bulgaria) and cultured in Tryptic Soy Broth (TSB; Biolife, Milan, Italy) at 28 °C on a rotary shaker at 197 rpm until complete sporulation. Following incubation for 72 h (or 5 days, depending on the experiment), spores were harvested by centrifugation at 6000 rpm and 4 °C for 15 min and washed twice with sterile distilled water. The final spore suspension was adjusted to 1 × 10^9^ spores/mL.

Fungal strains *Fusarium avenaceum* and *Alternaria solani* were also obtained from Biodinamika Ltd. (Plovdiv, Bulgaria) and maintained on Potato Dextrose Agar (PDA; Merck, Darmstadt, Germany). For inoculation, 5 mm diameter agar plugs containing actively growing mycelium were placed at the center of fresh PDA plates, which were then incubated at 28 °C until full mycelial growth was achieved. Under these conditions, *F. avenaceum* cultures additionally produced conidiospores.

### 2.2. Solution Casting of Chitosan Films

Chitosan-based films, both with and without encapsulated bacteria, were prepared using the solution casting method. Film-forming solutions were prepared at a concentration of 1% (*w*/*v*). CS-LMW, CS-MMW, and CS-HMW powders were first dispersed in distilled water under continuous magnetic stirring at room temperature to ensure proper wetting of the polymer particles. Subsequently, glacial acetic acid was added dropwise until complete dissolution of chitosan was achieved, resulting in clear homogeneous solutions. In contrast, COS was dissolved directly in distilled water without the addition of acid due to its inherent water solubility. Equal volumes of each solution (COS, CS-LMW, CS-MMW, and CS-HMW) were poured into Petri dishes and allowed to dry at room temperature under ambient conditions until constant weight was reached, forming uniform films. All films were cast under identical laboratory conditions at room temperature on a leveled surface to ensure uniform spreading of the polymer solution during drying.

For bacterial encapsulation, the appropriate amount of *Bacillus subtilis* spore suspension was added to the respective polymer solutions prior to casting. A ratio of 5 mL of bacterial suspension per 1 g of chitosan was used to achieve the desired microbial loading. This ratio provided a stable film-forming system and enabled uniform distribution of *B. subtilis* spores within the chitosan matrix. The bacterial-chitosan solutions were gently mixed to ensure homogeneous distribution of the spores throughout the film matrix prior to casting and drying.

### 2.3. Characterization

The surface morphology of the films was examined using a scanning electron microscope (JSM-5510, JEOL Co., Ltd., Tokyo, Japan). Prior to imaging, the samples were sputter-coated with a thin gold layer (~5–10 nm) using a JEOL JFC-1200 fine coater to improve conductivity. Morphometric analysis of bacterial spores was performed based on SEM micrographs using ImageJ software (v. 1.54g). The dynamic viscosity of the prepared chitosan solutions was measured using a Brookfield DV-II+ Pro programmable viscometer (Brookfield Engineering Laboratories, Middleboro, MA, USA) equipped with a cone spindle (CPE-52) and a thermostated sample cup at 25 °C.

Mechanical properties, including tensile strength, elongation at break, and Young’s modulus, were determined using an Instron 3344 universal testing machine (Instron, Norwood, MA, USA) equipped with a 50 N load cell. Rectangular specimens (20 × 60 mm) were tested with a crosshead speed of 10 mm/min. Stress–strain curves were recorded using Bluehill Universal software (v. 3.11), and mechanical parameters were calculated from the initial linear region of the curves. Reported values represent the mean of at least ten independent measurements. Film thickness was measured using a digital thickness gauge (FD 50, Käfer GmbH, Bremen, Germany).

The swelling behavior of COS, CS-LMW, CS-MMW, and CS-HMW films was evaluated gravimetrically in buffer solutions at pH 4, 7, and 9 (ionic strength I = 0.1) at 25 °C. Pre-weighed dry square film samples were immersed in the respective buffer solutions and allowed to swell until equilibrium was reached. At predetermined time intervals, the samples were removed, gently blotted to eliminate excess surface liquid, and weighed. This procedure was repeated until a constant mass corresponding to the equilibrium swelling state was obtained. The experiment was terminated after 24 h, as a decrease in swelling degree was observed beyond this time. The swelling degree (S, %) was calculated using the following Equation (1):

(1) $$S,\;\%\;=\;\frac{weight\;of\;swollen\;film-weight\;of\;dry\;film}{weight\;of\;dry\;film}\times 100,$$

where the weights of the films in the swollen and initial dry states are used, respectively. All measurements were performed in triplicate, and the mean values were reported.

### 2.4. Microbiological Evaluation and Antifungal Activity

The viability and metabolic activity of *Bacillus subtilis* encapsulated within COS and chitosan-based films were evaluated based on their ability to germinate and form colonies on solid nutrient medium. Tryptic Soy Agar (TSA) plates were prepared and sterilized prior to use. Circular film disks (10 mm diameter) containing bacterial spores were aseptically placed onto the surface of TSA and incubated at 28 °C. Bacterial growth and colony development were monitored after 48 h to assess spore germination and proliferation. Chitosan-based films without bacterial incorporation were used as controls to confirm sterility and exclude contamination.

The antifungal activity of the films was assessed using a dual-culture (spot-on-lawn) assay against the phytopathogenic fungi *Fusarium avenaceum* and *Alternaria solani*. A fungal suspension (10^3^ spores/mL) was uniformly spread onto Potato Dextrose Agar (PDA) plates and allowed to dry under sterile conditions. Subsequently, 10 mm disks of the test materials (with or without *B. subtilis*) were placed at the center of the inoculated plates. The plates were incubated at 28 °C for 5–7 days. Antagonistic activity was evaluated by measuring the diameter of the inhibition zone surrounding the film disks, corresponding to the area where fungal growth was suppressed. Radial fungal growth was also recorded to assess the extent of growth inhibition.

### 2.5. Statistical Analysis

Statistical analyses were performed using GraphPad Prism (v. 5.0, GraphPad Software Inc., San Diego, CA, USA). Data are presented as mean ± standard deviation (SD). The SD was calculated as the sample standard deviation because the measurements were performed using five independent replicates for each formulation (n = 5). The SD was calculated according to the following Equation (2):

(2) $$s\;=\;\sqrt{\frac{{\Sigma (x_{i}-\bar{x})}^{2}}{n-1}},$$

where x_i_ represents the individual values of the measured inhibition zones (fungicidal for *A. solani* and fungistatic for *F. avenaceum*), $\bar{x}$ is the arithmetic mean of the respective measurements, and (n − 1) represents the degrees of freedom. The use of (n − 1) corresponds to Bessel’s correction, which provides a more accurate estimate of variability for small sample sizes.

Differences among groups were evaluated by one-way analysis of variance (ANOVA) followed by Bonferroni’s post hoc test. Statistical significance was defined as follows: *p* < 0.05 (*), *p* < 0.01 (**), and *p* < 0.001 (***).

## 3. Results

The present study focuses on chitosan-based film matrices and systematically evaluates the influence of chitosan molecular weight on morphology, swelling behavior, mechanical properties, and microbial compatibility. The primary objective was to develop chitosan films as carriers for *Bacillus subtilis* and to assess their suitability as bioactive materials for plant protection. Particular emphasis was placed on understanding how polymer molecular weight influences film structure and the biological performance of the encapsulated microorganism.

Film-forming solutions were prepared at a concentration of 1% (*w*/*v*) for all molecular weight variants. CS-LMW, CS-MMW, and CS-HMW were dissolved in dilute acetic acid, whereas COS was dissolved directly in distilled water due to its inherent water solubility. This concentration ensured suitable viscosity for solvent casting and enabled reproducible formation of uniform, continuous, and self-supporting films after drying.

Transparent chitosan-based films were successfully prepared by solvent casting. The obtained films were visually homogeneous, smooth, and free of visible defects, exhibiting good optical transparency, uniform thickness, and sufficient mechanical integrity for handling and cutting into discs for microbiological assays. This observation is consistent with the mechanical testing results presented below, which demonstrate that the films possessed sufficient tensile strength and elasticity to maintain structural integrity during handling and sample preparation.

Film thickness was consistent within each formulation, confirming reproducibility of the casting process. Considering the identical polymer concentration (1%) and varying molecular weights, film thickness increased in the following order: COS (34 ± 1.5 µm) < CS-LMW (36 ± 1.8 µm) < CS-MMW (43 ± 2.1 µm) < CS-HMW (57 ± 2.3 µm). The ability to form continuous, self-supporting films without additional cross-linkers demonstrates the intrinsic film-forming capacity of chitosan across different molecular weights.

Prior to casting, the rheological properties of the four chitosan solutions (COS, CS-LMW, CS-MMW, and CS-HMW) were evaluated.

Dynamic viscosity measurements confirmed a strong dependence on molecular weight, with values of 73, 218, 480, and 614 cP for COS, CS-LMW, CS-MMW, and CS-HMW, respectively. The progressive increase in viscosity reflects enhanced chain length and intermolecular entanglement, which subsequently influence film thickness and microstructure, consistent with previous reports [31,32].

### 3.1. Morphological Characterization of Chitosan Films

**Figure 1:**
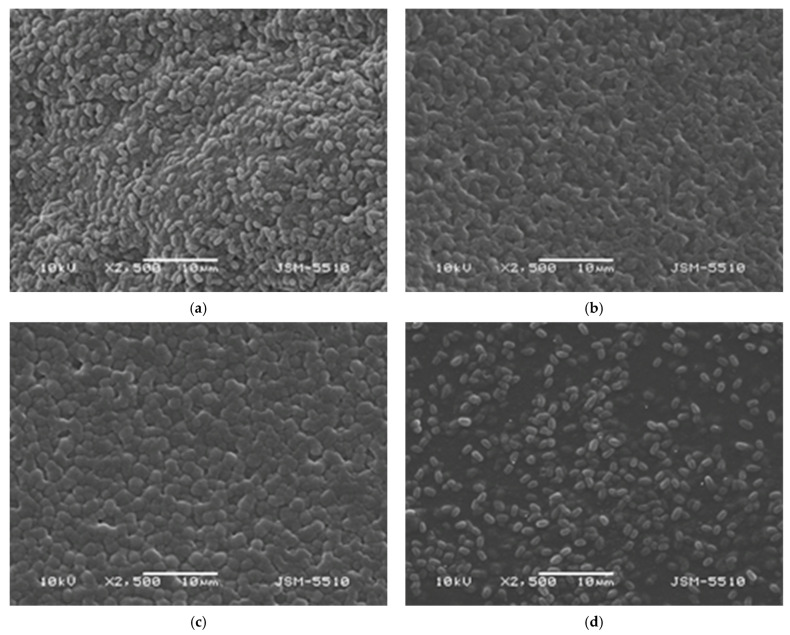
SEM micrographs of chitosan-based films loaded with *Bacillus subtilis*: (**a**) COS; (**b**) CS-LMW; (**c**) CS-MMW; (**d**) CS-HMW.

Scanning electron microscopy (SEM) was used to evaluate the surface morphology of the chitosan-based films before and after incorporation of *Bacillus subtilis*. SEM micrographs of the bacteria-loaded films (Figure 1) reveal homogeneous dispersion of rod-shaped *B. subtilis* spores within all polymer matrices (COS, CS-LMW, CS-MMW, and CS-HMW). The bacterial spores are clearly distinguishable and uniformly distributed, with no evidence of aggregation or clustering. Importantly, the characteristic rod-like morphology of *B. subtilis* (length 1.48 ± 0.17 µm and width 0.73 ± 0.89 µm) was preserved, indicating that neither acidic dissolution nor solvent-casting and drying steps compromised cellular structural integrity.

A progressive increase in surface compactness was observed with increasing chitosan molecular weight. COS-based films (Figure 1a) exhibited a comparatively less compact and slightly more open surface morphology, which can be attributed to the shorter chain length and lower degree of intermolecular entanglement of COS. In contrast, CS-LMW and CS-MMW films (Figure 1b,c) showed improved surface continuity and denser microstructural organization. CS-HMW films (Figure 1d) displayed the most compact and continuous morphology, consistent with enhanced chain entanglement and stronger intermolecular hydrogen bonding during solvent evaporation.

Compared to pristine films (Figure S1, Supplementary Materials), bacteria-loaded films exhibited slightly increased surface roughness due to embedded spores and localized polymer–*B. subtilis* interactions. Nevertheless, no cracks, phase separation, or structural defects were detected, confirming good film-forming ability and structural integrity across all molecular weight variants. SEM analysis of pristine COS and chitosan-based films revealed smooth, homogeneous, and defect-free surfaces, further supporting the formation of continuous polymer networks prior to bacterial incorporation.

### 3.2. Swelling Behavior and Structural Stability of Chitosan Films

When exposed to water droplets, the films exhibited rapid surface hydration and gel-like swelling, confirming their hydrophilic character and ability to interact with aqueous environments—an important feature for nutrient diffusion and microbial viability.

**Figure 2:**
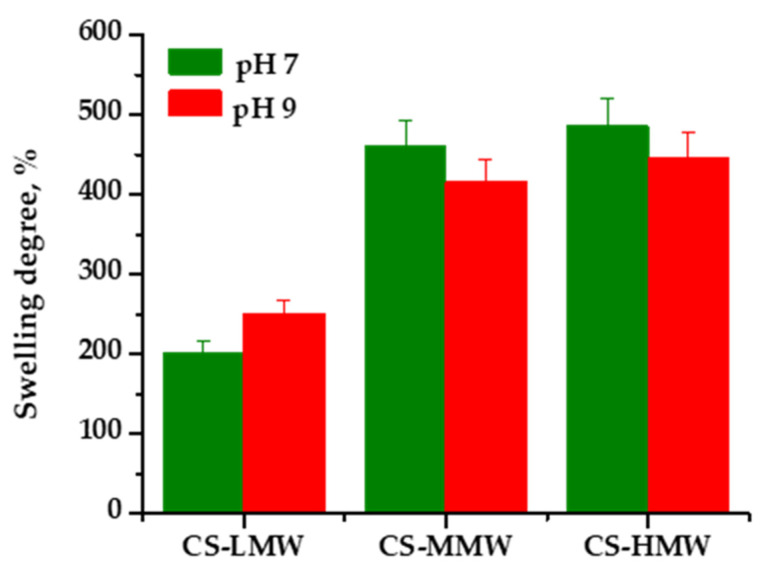
Swelling degree of chitosan-based films at 25 °C as a function of pH. Legend: pH 7—green; pH 9—red.

The swelling behavior was evaluated at pH 4, 7, and 9 (Figure 2). As expected, COS dissolved completely under all tested pH conditions. This is attributed to its water solubility, lower molecular weight and short chain length, which prevent the formation of a physically entangled polymer network. The lack of sufficient intermolecular interactions results in complete solubilization rather than swelling, independent of pH. Similarly, at pH 4, all chitosan films dissolved within 15 min due to protonation of amino groups (pKa ≈ 6.5) [22], which generated electrostatic repulsion between chains and disrupted the hydrogen-bonding network. This confirms the typical polyelectrolyte behavior of chitosan under acidic conditions and the absence of covalent crosslinking.

In contrast, at pH 7, all chitosan films maintained structural integrity and reached equilibrium swelling (Figure 2). A clear molecular weight dependence was observed: swelling increased with increasing molecular weight. Higher molecular weight films formed more entangled networks capable of retaining larger amounts of water while maintaining structural stability. The relatively small difference between CS-MMW and CS-HMW suggests the presence of a molecular weight threshold beyond which further entanglement provides diminishing gains in water uptake.

At pH 9, CS-LMW showed a moderate increase in swelling, while CS-MMW and CS-HMW exhibited slightly reduced swelling compared to neutral conditions (Figure 2). Under alkaline conditions, deprotonation of amino groups reduces electrostatic repulsion and promotes intermolecular hydrogen bonding and hydrophobic interactions, leading to network compaction in higher molecular weight films.

These findings indicate that molecular weight governs not only water uptake capacity but also structural stability under varying pH conditions.

### 3.3. Mechanical Properties of Chitosan Films

**Figure 3:**
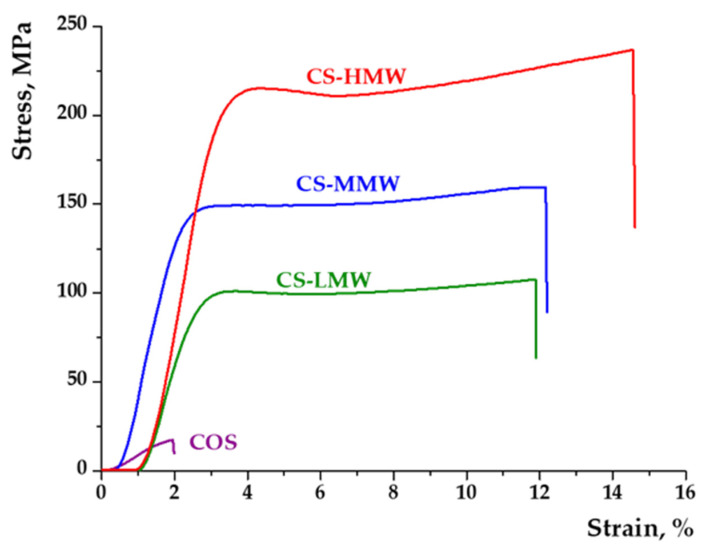
Stress–strain curves of chitosan-based films. Legend: COS—violet line; CS-LMW—green line; CS-MMW—blue line; CS-HMW—red line.

Mechanical characterization further confirmed the dominant influence of molecular weight on film performance (Figure 3). COS films exhibited brittle behavior, failing at ~1–2% strain and reaching maximum stress below 15 MPa, reflecting insufficient chain entanglement. CS-LMW films showed significantly improved properties (tensile strength~100 MPa; elongation at break~10%). CS-MMW films demonstrated further enhancement (~150 MPa; ~12–13% strain), consistent with increased intermolecular interactions and plastic deformation capacity. CS-HMW films exhibited the highest tensile strength (>200 MPa) and elongation (~15–16%), indicating a highly entangled and mechanically robust polymer network. The mechanical trends correlate directly with SEM and swelling results: increasing molecular weight leads to denser morphology, higher entanglement density, improved structural stability, and enhanced resistance to deformation.

SEM, swelling, and mechanical analyses demonstrate that polymer molecular weight is the key parameter controlling network architecture, hydration behavior, and mechanical resilience of the films. High molecular weight chitosans form compact, entangled matrices that maintain integrity under neutral and alkaline conditions while providing superior mechanical strength. Importantly, this structurally robust yet hydrophilic network enables uniform encapsulation of *Bacillus subtilis*, supporting bacterial structural preservation and creating a favorable microenvironment for sustained biological activity. The combined structural, physicochemical, and biological performance highlights the potential of these films as effective bioactive carriers for sustainable plant protection.

### 3.4. Effect of Chitosan Molecular Weight on Bacillus subtilis Viability and Stability

**Figure 4:**
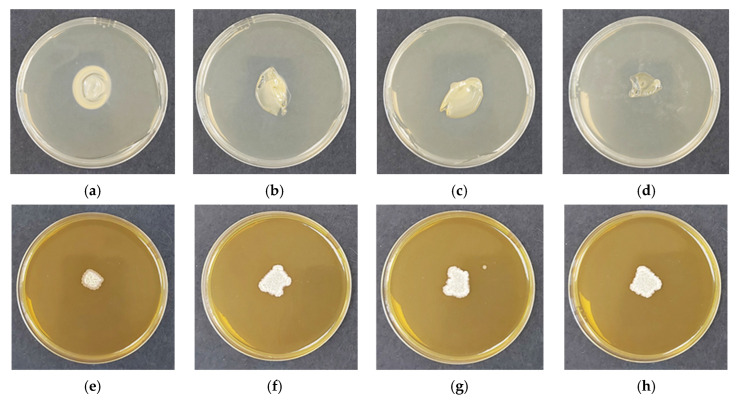
Digital images of chitosan-based films after 48 h of incubation. Bacteria-free films: (**a**) COS; (**b**) CS-LMW; (**c**) CS-MMW; (**d**) CS-HMW. (**e**–**h**) Films containing *Bacillus subtilis*: (**e**) COS/*B. subtilis*; (**f**) CS-LMW/*B. subtilis*; (**g**) CS-MMW/*B. subtilis*; (**h**) CS-HMW/*B. subtilis*. Panels (**a**–**d**) show biological purity, while panels (**e**–**h**) show bacterial viability.

The ability of *Bacillus subtilis* to survive, germinate, and complete its vegetative development after encapsulation into chitosan-based films was evaluated. Biological purity was first verified using control films without encapsulated spores. These samples were incubated under identical conditions, and no unintended microbial growth was observed (Figure 4a–d), demonstrating sterility of the films and the absence of contaminating microflora.

When placed onto Tryptic Soy Agar (TSA), all film formulations absorbed moisture and underwent hydration-driven swelling (Figure 4). However, the extent of swelling and interfacial mass transfer varied markedly with chitosan molecular weight. COS-, CS-LMW-, and CS-MMW-based films formed visible interaction zones beneath the film–agar interface. This phenomenon was most pronounced for COS, where radial diffusion beyond the initial contact area was clearly observed. The enhanced spreading behavior can be attributed to the low molecular weight and high chain mobility of COS, which facilitates diffusion into the agar matrix. Within these regions, a distinct interfacial precipitate was detected, likely resulting from ionic interactions between protonated amino groups of chitosan and negatively charged components of the agar medium. The boundaries of these interaction zones remained well defined throughout incubation. In contrast, CS-HMW films preserved greater structural cohesion and exhibited more localized hydration. The denser and more highly entangled polymer network characteristic of high-molecular-weight chitosan likely restricts extensive diffusion into the substrate, thereby concentrating interactions near the contact interface.

Following verification of sterility, the viability of encapsulated *B. subtilis* was assessed. Upon placement on nutrient agar, the films rapidly absorbed moisture, generating a hydrated microenvironment conducive to spore germination. After 48 h of incubation, visible bacterial colonies were observed for all formulations (Figure 4e–h), confirming that neither acidic processing nor solvent casting compromised spore viability.

Although viability was maintained across all molecular weight variants, differences in colony morphology and spatial expansion were evident. COS-based films (Figure 4e) produced relatively compact colonies with limited radial spread compared to the other formulations. In contrast, CS-LMW, CS-MMW, and CS-HMW films (Figure 4f–h) exhibited broader and more irregular colony expansion, with no pronounced differences observed among these three molecular weight variants. The colonies in these matrices appeared similarly distributed, suggesting comparable hydration behavior and nutrient accessibility once the films were in contact with the agar medium. These observations indicate that the primary distinction in microenvironmental modulation occurs between the oligomeric COS system and the polymeric chitosan matrices rather than progressively across increasing molecular weights. The water-soluble nature of COS likely promotes rapid swelling and partial diffusion into the agar substrate, which may alter local nutrient gradients and spatially confine bacterial growth. In contrast, the higher molecular weight chitosans (CS-LMW, CS-MMW, and CS-HMW) form more structurally stable films that maintain consistent interfacial contact with the substrate, supporting similar mass transfer conditions and resulting in denser and more spatially confined colony growth.

Importantly, despite these morphological variations, no inhibition of germination or vegetative growth was detected in any formulation, confirming that molecular weight influences colony morphology primarily through physicochemical modulation of the microenvironment rather than through direct effects on bacterial viability. The developed chitosan films therefore function as biologically safe and structurally stable carriers, preserving microbial activity while maintaining sterility—an essential requirement for sustainable plant protection applications.

### 3.5. Effect of Chitosan Molecular Weight on Antifungal Activity Against Plant Pathogens

**Figure 5:**
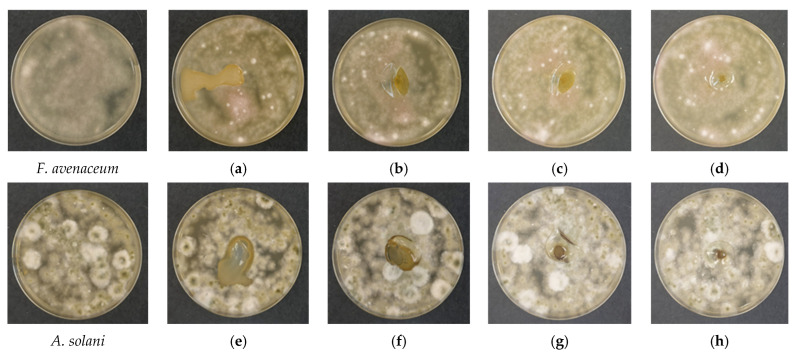
Antifungal activity of bacteria-free chitosan films after 48 h of incubation. (**a**–**d**) In the presence of *Fusarium avenaceum*: (**a**) COS; (**b**) CS-LMW; (**c**) CS-MMW; (**d**) CS-HMW. (**e**–**h**) In the presence of *Alternaria solani*: (**e**) COS; (**f**) CS-LMW; (**g**) CS-MMW; (**h**) CS-HMW.

The antifungal activity of the developed chitosan films was evaluated using a spot-on-lawn assay against *Fusarium avenaceum* and *Alternaria solani*, enabling simultaneous pathogen development and direct interaction with the polymer matrix. A comparative analysis of bacteria-free chitosan films revealed that antifungal performance is governed not only by intrinsic chitosan activity but also by molecular weight-dependent diffusion behavior within the agar substrate. After 48 h of incubation, control plates exhibited uniform and dense mycelial growth of *F. avenaceum* (Figure 5), confirming optimal culture conditions. In contrast, chitosan-based films demonstrated moderate but reproducible growth inhibition, the extent and spatial pattern of which strongly depended on polymer molecular weight.

A key observation concerns the COS-based formulation (Figure 5a,e). Owing to its water solubility and high chain mobility, COS films exhibited pronounced radial diffusion into the agar, forming an extended interaction zone. This diffusion pattern significantly influenced pathogen response, particularly for *A. solani*. A distinct non-contact inhibition halo was observed, where fungal growth was arrested at the outer boundary of the COS diffusion zone without direct contact with the material. This indicates that diffusible chitosan oligomers migrated into the substrate and exerted fungistatic effects at a distance. The previously observed precipitated interfacial region likely reflects ionic interactions between COS and medium components, potentially modifying the local microenvironment and contributing to growth suppression. In contrast, *F. avenaceum* responded differently to COS. Although fungal hyphae did not colonize the film surface, growth progressed up to the material boundary, and no pronounced non-contact inhibition zone was detected. This suggests species-specific susceptibility, with *F. avenaceum* displaying greater tolerance to diffusible oligomeric fractions compared to *A. solani*.

For higher-molecular-weight formulations (CS-LMW, CS-MMW, CS-HMW), the antifungal mechanism appeared fundamentally different. These films maintained structural integrity and showed limited radial diffusion into the agar. In some cases, incomplete hydration was observed, manifested by partial lifting of the films from the agar surface as fungal growth progressed beneath or around the material. This behavior indicates reduced interfacial mass transfer and less efficient diffusion of active polymer fractions into the substrate. Under these conditions, antifungal activity was largely confined to direct contact zones. For example, in the CS-LMW/*A. solani* (Figure 5f) system, adequate hydration allowed close interfacial interaction, and fungal growth was halted at the film boundary without penetration beneath the film. However, unlike COS, no distant inhibition halo was formed.

Overall, these findings demonstrate that antifungal efficacy in this assay depends critically on diffusion capacity and substrate interaction rather than solely on polymer density. COS exhibited superior spatial antifungal performance against *A. solani*, attributable to the bioavailability of mobile oligomeric chains capable of modifying the surrounding medium. Conversely, higher-molecular-weight chitosans function predominantly as localized physical and chemical barriers, with their effectiveness strongly influenced by hydration and contact efficiency.

Importantly, under the specific conditions of the spot-on-lawn assay, where inoculum density and diffusion dynamics influence outcomes, COS displayed the highest intrinsic antifungal activity against *A. solani* among the bacteria-free formulations. These results highlight that molecular weight governs not only the structural properties of chitosan films but also the dominant antifungal mechanism, shifting from diffusion-mediated inhibition (COS) to contact-dependent barrier effects (CS-LMW–CS-HMW).

### 3.6. Antifungal Activity of Biohybrid Chitosan Films

The antifungal efficacy of the developed biohybrid films containing encapsulated *Bacillus subtilis* spores was comprehensively evaluated using a spot-on-lawn assay against *Fusarium avenaceum* and *Alternaria solani*. This model was selected over conventional dual-culture assays because it closely simulates direct-contact conditions relevant to practical agricultural applications. In this configuration, the polymer film is placed directly onto a uniform fungal lawn, enabling immediate interfacial interaction, synchronous swelling of the matrix, and simultaneous germination of bacterial spores and fungal propagules.

Upon contact with the inoculated agar, the biohybrid films rapidly absorbed moisture and swelled, generating a hydrated interfacial microenvironment that functioned as a localized “micro-bioreactor”. Within this dynamic interface, three parallel antifungal mechanisms operated simultaneously: (i) direct electrostatic stress exerted by protonated chitosan chains on negatively charged fungal cell membranes; (ii) nutrient competition between proliferating *B. subtilis* cells and the pathogen; and (iii) active biological protection through biofilm establishment and controlled synthesis and release of antifungal metabolites.

Quantitative analysis demonstrated that antifungal efficacy was strongly dependent on chitosan molecular weight (Table 1). The antifungal activity was evaluated in quintuplicate (n = 5), and the results are presented as mean values ± standard deviation (SD). The SD was calculated using the sample standard deviation formula with Bessel’s correction (n − 1), providing a more accurate estimate for the relatively small sample size and ensuring that variability within each experimental group is appropriately reflected.

**Figure 6:**
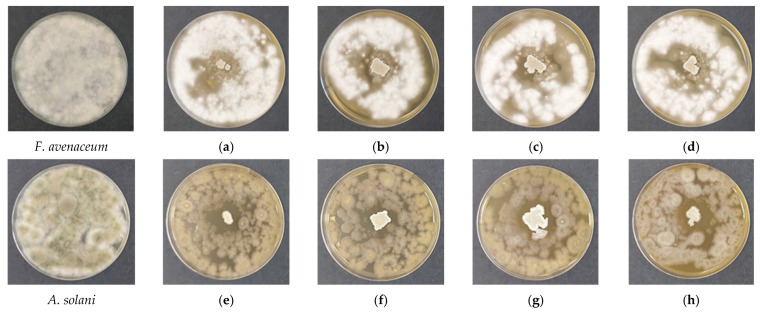
Antifungal activity of biohybrid chitosan films containing *Bacillus subtilis* after 48 h of incubation. (**a**–**d**) In the presence of *Fusarium avenaceum*: (**a**) COS/*B. subtilis*; (**b**) CS-LMW/*B. subtilis*; (**c**) CS-MMW/*B. subtilis*; (**d**) CS-HMW/*B. subtilis*. (**e**–**h**) In the presence of *Alternaria solani*: (**e**) COS/*B. subtilis*; (**f**) CS-LMW/*B. subtilis*; (**g**) CS-MMW/*B. subtilis*; (**h**) CS-HMW/*B. subtilis*.

For *F. avenaceum*, inhibition was predominantly fungistatic, and no clearly separated sterile zone was observed, reflecting the relatively high tolerance of this pathogen (Figure 6a–d). Consequently, the measured values correspond to the external fungistatic inhibition zone (Table 1). One-way ANOVA revealed significant differences among the molecular weight variants (*p* < 0.05). According to Tukey’s grouping, CS-MMW (32.6 ± 1.8 mm) and CS-HMW (31.6 ± 1.5 mm) exhibited the highest inhibitory capacity, followed by CS-LMW (29.4 ± 1.1 mm), while COS showed the lowest activity (27.4 ± 2.9 mm). These findings indicate that, for more resilient pathogens, structural stability of the matrix plays a more decisive role than rapid oligomer diffusion. Longer polymer chains provide enhanced network cohesion, improved bacterial retention, and sustained metabolite accumulation at the interface, thereby strengthening the fungistatic effect.

In contrast, *A. solani* exhibited a distinct dual-zone inhibition pattern (Figure 6e–h). For this pathogen, the fungicidal internal zone was considered the primary indicator of antifungal efficacy (Table 1). While the internal zone showed comparable activity across formulations, the external fungistatic halo displayed a strong dependence on molecular weight (*p* < 0.001). CS-HMW produced the most extensive inhibition zone (84.6 ± 5.0 mm), significantly exceeding all other variants. COS showed intermediate activity (64.0 ± 3.2 mm), whereas CS-LMW (57.6 ± 2.7 mm) and CS-MMW (59.3 ± 1.1 mm) formed a statistically similar lower-activity group. This pronounced “halo effect” observed in the CS-HMW system (Figure 6h) is attributed to enhanced stabilization of the bacterial population and modulation of mass-transfer processes within the denser polymer network. The cohesive matrix likely regulates the diffusion of antifungal metabolites, creating a sustained suppressive environment extending beyond the physical boundary of the film.

The relatively low SD values observed across the datasets (Table 1) demonstrate the high reproducibility of the solvent-casting method used for film preparation. In particular, the CS-HMW formulation showed the most consistent and potent activity against both pathogens, with especially high precision in the fungistatic effect against *F. avenaceum*. The distinct fungicidal zones observed for both pathogens suggest that bioactive metabolites produced by the encapsulated *B. subtilis* possess strong membrane-disruptive potential (Figure 6). This effect may range from complete degradation of fungal cellular structures to severe physiological suppression, with the intensity of the response varying according to the adaptive capacity of the specific pathogen.

These results demonstrate that the efficacy of biohybrid chitosan systems is pathogen-dependent and must be strategically calibrated. While COS offers advantages associated with rapid diffusion of oligomeric chains, high-molecular-weight chitosans provide superior structural integrity, improved biofilm stabilization, and controlled metabolite release—features that are critical for sustainable plant protection applications.

The antifungal performance of the biohybrid films arises from multiple complementary mechanisms. Chitosan exerts primary chemical stress through electrostatic interactions between protonated amino groups and negatively charged fungal cell wall components, increasing membrane permeability and impairing nutrient uptake. Simultaneously, the hydrated polymer matrix functions as a semi-permeable barrier, modulating local mass transfer. In the biohybrid systems, the matrix additionally regulates the spatial distribution and persistence of bacterial metabolites. Higher-molecular-weight chitosan forms a cohesive network that enhances bacterial retention and stabilizes the biofilm, promoting sustained antifungal activity and gradual metabolite release.

The relative contributions of these mechanisms are pathogen-dependent. For *A. solani*, the expansive inhibition zone of CS-HMW films highlights the importance of matrix stability and controlled metabolite release. For *F. avenaceum*, which is intrinsically more resistant, structural robustness of the matrix (CS-MMW/CS-HMW) is more critical than oligomer diffusion.

Overall, these results demonstrate that antifungal efficacy in biohybrid chitosan films is governed by a complex interplay of polymer molecular weight, swelling behavior, matrix architecture, bacterial biofilm formation, and pathogen-specific sensitivity. While COS facilitates rapid diffusion, higher-molecular-weight chitosans provide superior performance against more resistant pathogens by stabilizing bacterial activity and controlling metabolite release. These findings underscore the importance of tailoring chitosan molecular weight to the target pathogen when designing biohybrid films for sustainable plant protection.

## 4. Discussion

The present study demonstrates that chitosan molecular weight is a key design parameter governing both the physicochemical behavior of the polymer matrix and its biological performance as a microbial carrier. The observed increase in solution viscosity, film thickness, and surface compactness with increasing molecular weight is consistent with the well-established relationship between polymer chain length, intermolecular entanglement, and hydrogen bonding in chitosan systems reported in previous studies [31,32]. These structural changes directly influence hydration dynamics and interfacial interactions, confirming that polymer architecture controls the functional properties of chitosan films.

The pronounced pH-responsive swelling behavior observed across all formulations reflects the polyelectrolyte nature of chitosan, driven by protonation of amino groups under acidic conditions. Molecular weight significantly modulated both the extent and kinetics of hydration. COS, due to its short chain length and high mobility, exhibited enhanced diffusion into the surrounding medium, whereas CS-LMW, CS-MMW, and CS-HMW formed more cohesive networks with restricted mass transfer. Similar relationships between molecular weight, swelling behavior, and diffusion characteristics have been described for chitosan-based materials used in controlled-release and antimicrobial applications [33,34,35].

In bacteria-free systems, COS exhibited a clear diffusion-mediated antifungal effect, particularly against *Alternaria solani*, where distinct non-contact inhibition zones were observed. This behavior can be attributed to the high solubility and mobility of oligomeric chitosan chains, which enable radial diffusion into the agar medium. Such diffusible fractions are known to interact with fungal cell walls, increasing membrane permeability and disrupting cellular homeostasis through electrostatic interactions and oxidative stress induction [23,33]. In contrast, higher-molecular-weight chitosans (CS-LMW, CS-MMW, and CS-HMW) maintained stronger structural integrity and acted predominantly through contact-dependent interfacial mechanisms, with antifungal activity largely confined to the film boundary. These findings indicate a molecular-weight-dependent transition from diffusion-driven inhibition in COS systems to structurally mediated interfacial effects in higher-molecular-weight matrices.

In biohybrid systems, the antifungal mechanism changed substantially due to the presence of encapsulated *Bacillus subtilis*. Antifungal performance resulted from the synergistic interaction between the chitosan matrix and bacterial metabolism. Chitosan provides a semi-permeable and protective microenvironment that supports bacterial survival while simultaneously contributing intrinsic antimicrobial activity. Similar synergistic effects between chitosan matrices and beneficial microbes have been reported in previous studies involving chitosan-based carriers for plant-protective bacteria [36,37].

Although all formulations maintained bacterial viability, higher-molecular-weight matrices provided superior structural stability, which likely improved bacterial retention, biofilm persistence, and localized accumulation of antifungal metabolites. This effect became particularly evident for *Fusarium avenaceum*, where inhibition was predominantly fungistatic and no clearly defined sterile zone was detected. The comparatively higher tolerance of *F. avenaceum* to the polymer–bacterial system suggests that matrix robustness and sustained metabolite retention play a more decisive role than rapid diffusion of oligomers. Longer polymer chains improve network cohesion and stabilize the interfacial microenvironment, thereby prolonging growth suppression.

In contrast, *Alternaria solani* exhibited a dual-zone inhibition pattern strongly dependent on molecular weight. While the internal sterile region did not differ significantly among formulations, the external fungistatic halo expanded markedly in the CS-HMW film. The large inhibition zone formed by CS-HMW suggests that controlled metabolite diffusion and stable biofilm organization within the denser matrix create a sustained suppressive microenvironment extending beyond the physical boundary of the film.

The antifungal activity observed in this study is consistent with previously reported inhibitory effects of both chitosan and *B. subtilis* against phytopathogenic fungi. Chitosan has been widely reported to inhibit fungal growth through electrostatic interactions with cell wall components, disruption of membrane permeability, and induction of oxidative stress [23,33,34,36]. Likewise, *B. subtilis* is known to produce a broad spectrum of antifungal metabolites, which contribute to pathogen suppression in plant-associated environments [38,39].

Recent research highlights the potential of chitosan–microbe composites for enhanced antifungal performance, but systematic tuning of chitosan molecular weight in cast film matrices remains underexplored. For example, Krastev et al. [30] reported that chitosan gel beads enhanced *B. subtilis* viability and pathogen suppression against *Fusarium* spp., demonstrating improved stability and microbial activity under stress conditions, but did not evaluate the influence of polymer chain length on performance. Similarly, Qiu et al. [37] showed that combined application of chitosan with *B. subtilis* significantly reduced disease incidence in greenhouse trials by modulating rhizosphere microbial communities and enhancing plant defense responses, yet this was achieved in soil amendments rather than structured film carriers. Furthermore, Zhang et al. [36] reviewed recent advances in chitosan’s antifungal mechanisms, underscoring the role of molecular weight and degree of deacetylation in governing polymer–pathogen interactions and transport properties. These studies collectively suggest that although chitosan and *B. subtilis* synergize for biocontrol, few reports have systematically integrated molecular-weight-dependent film architecture with bacterial encapsulation and antifungal performance, particularly in a cast film format. By addressing this gap, the present work provides a quantitative comparison of structural, diffusion, and biological effects across chitosan films of different molecular weights, revealing a mechanistic shift in antifungal action that depends on both polymer architecture and pathogen sensitivity. This advances the field beyond existing research and offers practical insights for the rational design of biohybrid plant protection materials.

These results reveal that molecular weight governs a mechanistic shift in antifungal action, from oligomer diffusion in bacteria-free systems to matrix-regulated microbial activity in biohybrid films. The relative importance of diffusion versus structural stabilization appears to be pathogen-dependent: *A. solani* responds strongly to sustained metabolite gradients, whereas *F. avenaceum* requires enhanced matrix robustness to achieve effective suppression.

From an application perspective, these findings highlight the importance of tailoring chitosan molecular weight to the target pathogen and desired release profile. Lower-molecular-weight systems may be advantageous where rapid diffusion and immediate antifungal activity are required, whereas higher-molecular-weight matrices appear better suited for sustained, structurally supported biocontrol strategies. Future studies should focus on long-term stability under field-relevant conditions, quantitative analysis of bacterial metabolite release, and evaluation in planta to confirm agronomic performance.

## 5. Conclusions

This study demonstrates that chitosan molecular weight critically influences the structural, physicochemical, and biological performance of chitosan films developed as carriers for *Bacillus subtilis*. Although all films were prepared at the same polymer concentration (1%, *w*/*v*), increasing molecular weight resulted in higher solution viscosity, increased film thickness, enhanced microstructural compactness, and improved mechanical integrity.

In bacteria-free systems, antifungal activity was largely governed by diffusion behavior. COS exhibited pronounced radial diffusion and strong non-contact inhibition against *Alternaria solani*, indicating a diffusion-mediated antifungal mechanism driven by mobile oligomeric chains. In contrast, CS-LMW, CS-MMW, and CS-HMW films acted mainly through contact-dependent interfacial mechanisms associated with their more cohesive polymer networks.

In biohybrid formulations containing encapsulated *B. subtilis*, antifungal efficacy resulted from the synergistic interaction between matrix architecture and bacterial activity. Higher-molecular-weight chitosans provided improved stabilization of bacterial colonization and enhanced retention of diffusible metabolites, resulting in stronger suppression of *Fusarium avenaceum*, which exhibited predominantly fungistatic inhibition under the tested conditions.

Overall, the results demonstrate that tuning the molecular weight of chitosan provides an effective strategy to regulate both the physicochemical properties of the polymer matrix and the biological performance of encapsulated microorganisms. These findings highlight the potential of chitosan-based cast films as bioactive carriers for beneficial bacteria in sustainable plant protection systems.

## Figures and Tables

**Table 1:** Antifungal activity of biohybrid films containing *B. subtilis*, expressed as inhibition zone diameters (mm) ± SD (n = 5).

Film Type	*F. avenaceum* Inner Zone (mm)	*F. avenaceum* Outer Zone (mm)	*A. solani* Inner Zone (mm)	*A. solani* Outer Zone (mm)
COS/*B. subtilis*	27.4 ± 2.9	–	26.6 ± 1.1	64.0 ± 3.2
CS-LMW/*B. subtilis*	29.4 ± 1.1	–	25.4 ± 1.1	57.6 ± 2.7
CS-MMW/*B. subtilis*	32.6 ± 1.8	–	28.2 ± 1.3	59.3 ± 1.1
CS-HMW/*B. subtilis*	31.6 ± 1.5	–	32.2 ± 2.6	84.6 ± 5.0

## Data Availability

The original contributions presented in this study are included in the article/supplementary material. Further inquiries can be directed to the corresponding author.

## Supplementary Materials

The following supporting information can be downloaded at: https://www.mdpi.com/article/10.3390/polym18070784/s1, Figure S1: SEM micrographs of chitosan-based cast films: (a) COS; (b) CS-LMW; (c) CS-MMW; (d) CS-HMW.

## Abbreviations

The following abbreviations are used in this manuscript:

COS	Chitooligosaccharide
CS-LMW	Low-molecular-weight chitosan
CS-MMW	Medium-molecular-weight chitosan
CS-HMW	High-molecular-weight chitosan
