# Supplementary material for: Chitosan-Based Cast Films of Different Molecular Weights for Sustained Activity of Bacillus subtilis
Source: Polymers (Basel). 2026 Mar 24;18(7):784. doi: 10.3390/polym18070784 (PMC13074804; doi:10.3390/polym18070784)
Supplement: Supplementary file 1 [file polymers-18-00784-s001.zip › polymers-4197308-supplementary.pdf]

polymers

MDPI

Supplementary data

Chitosan-Based Cast Films of Different Molecular Weights for Sustained Activity of *Bacillus subtilis*

Vladimir Krastev [1], Nikoleta Stoyanova [1,2], Iliyana Valcheva [3], Donka Draganova [3], Mariya Spasova [1,2] and Olya Stoilova [1,*]

1 Laboratory of Bioactive Polymers, Institute of Polymers, Bulgarian Academy of Sciences, 1113 Sofia, Bulgaria; v_krastev@polymer.bas.bg (V.K.); nstoyanova@polymer.bas.bg (N.S.); mspasova@polymer.bas.bg (M.S.)
2 Centre of Competence "Sustainable Utilization of Bio-resources and Waste of Medicinal and Aromatic Plants for Innovative Bioactive Products" (CoC BioResources), 1000 Sofia, Bulgaria
2 Biodinamika Ltd., 4000 Plovdiv, Bulgaria; donkadraganova@gmail.com (D.D.), valchevailiana1@gmail.com (I.V.)
* Correspondence: stoilova@polymer.bas.bg

Received: date; Accepted: date; Published: date

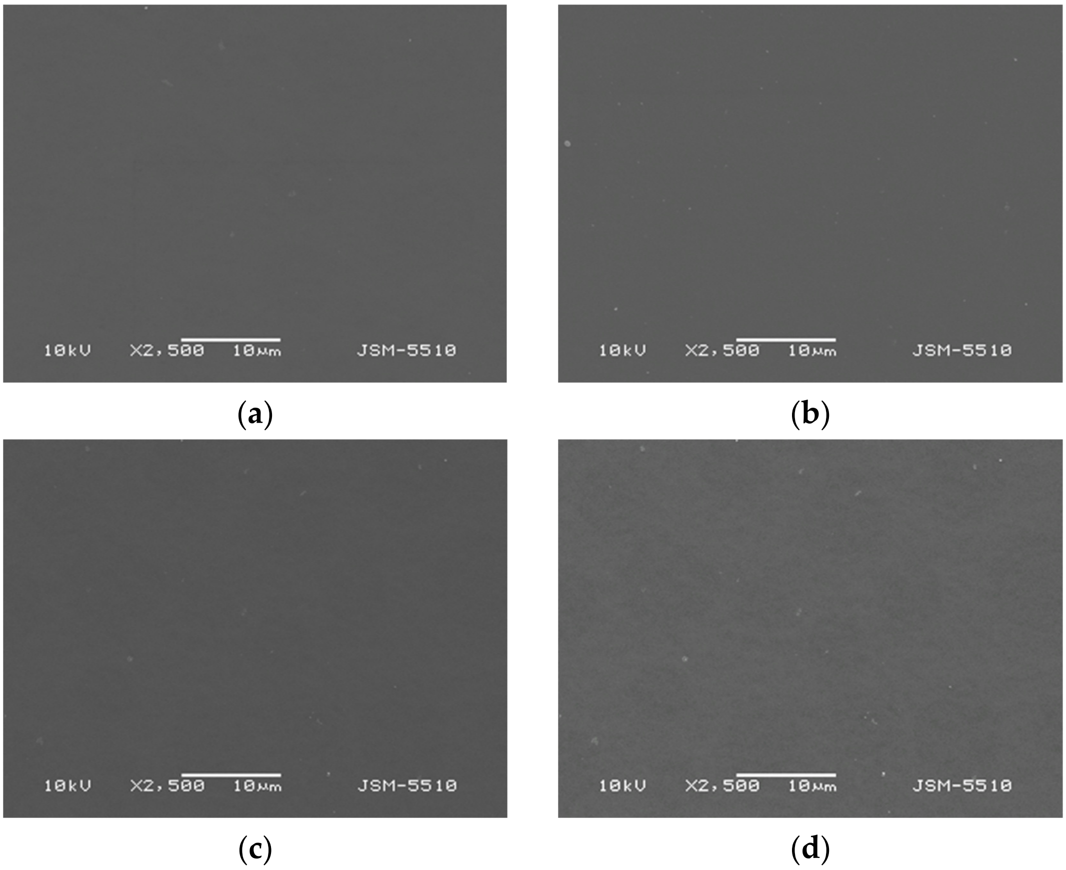

Figure S1. SEM micrographs of chitosan-based cast films: (**a**) COS; (**b**) CS-LMW; (**c**) CS-MMW; (**d**) CS-HMW.
